# From calcium imaging to graph topology

**DOI:** 10.1162/netn_a_00262

**Published:** 2022-10-01

**Authors:** Ann S. Blevins, Dani S. Bassett, Ethan K. Scott, Gilles C. Vanwalleghem

**Affiliations:** Department of Bioengineering, School of Engineering and Applied Science, University of Pennsylvania, Philadelphia, PA, USA; Department of Psychiatry, Perelman School of Medicine, University of Pennsylvania, Philadelphia, PA, USA; Department of Neurology, Perelman School of Medicine, University of Pennsylvania, Philadelphia, PA, USA; Department of Electrical and Systems Engineering, School of Engineering and Applied Science, University of Pennsylvania, Philadelphia, PA, USA; Department of Physics and Astronomy, College of Arts and Sciences, University of Pennsylvania, Philadelphia, PA, USA; Santa Fe Institute, Santa Fe, NM, USA; Queensland Brain Institute, University of Queensland, Brisbane, Australia; Department of Anatomy and Physiology, School of Biomedical Sciences, University of Melbourne, Parkville, Australia; Danish Research Institute of Translational Neuroscience (DANDRITE), Nordic EMBL Partnership for Molecular Medicine, Aarhus University, Aarhus, Denmark; Department of Molecular Biology and Genetics, Aarhus University, Aarhus, Denmark

**Keywords:** Zebrafish, Topology, Calcium imaging, Graph theory, Systems neuroscience

## Abstract

Systems neuroscience is facing an ever-growing mountain of data. Recent advances in protein engineering and microscopy have together led to a paradigm shift in neuroscience; using fluorescence, we can now image the activity of every neuron through the whole brain of behaving animals. Even in larger organisms, the number of neurons that we can record simultaneously is increasing exponentially with time. This increase in the dimensionality of the data is being met with an explosion of computational and mathematical methods, each using disparate terminology, distinct approaches, and diverse mathematical concepts. Here we collect, organize, and explain multiple data analysis techniques that have been, or could be, applied to whole-brain imaging, using larval zebrafish as an example model. We begin with methods such as linear regression that are designed to detect relations between two variables. Next, we progress through network science and applied topological methods, which focus on the patterns of relations among many variables. Finally, we highlight the potential of generative models that could provide testable hypotheses on wiring rules and network progression through time, or disease progression. While we use examples of imaging from larval zebrafish, these approaches are suitable for any population-scale neural network modeling, and indeed, to applications beyond systems neuroscience. Computational approaches from network science and applied topology are not limited to larval zebrafish, or even to systems neuroscience, and we therefore conclude with a discussion of how such methods can be applied to diverse problems across the biological sciences.

## INTRODUCTION

**Figure 1.**
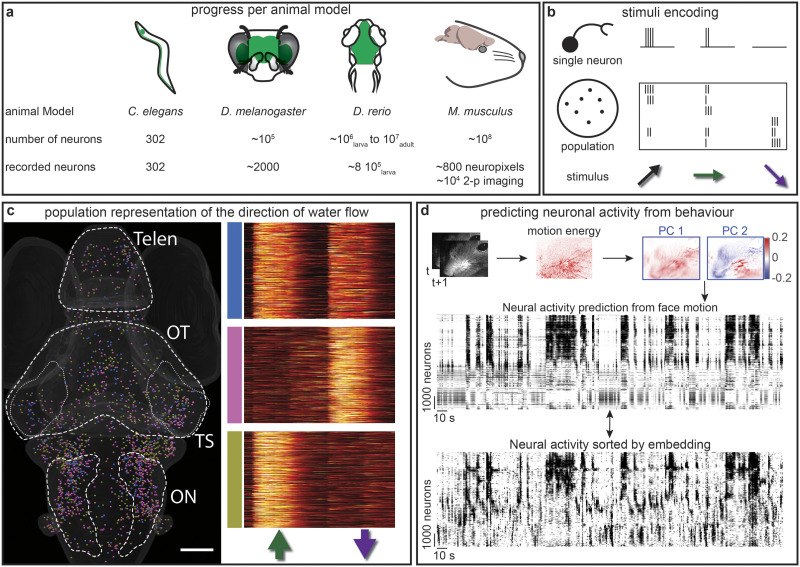
Rapid progress of systems neuroscience and the encoding of stimuli. (A) Across animal models of increasing complexity, we are now able to record the activity of many neurons. For example, we can record all of the neurons of *C. elegans*, about 2,000 neurons of *D. melanogaster* (Tainton-Heap et al., 2021), and about 80% of neurons in the larval zebrafish (*D. rerio*; X. Chen et al., 2018). High-density electrophysiological probes allow the recording of hundreds of units per session in mice, including deep structures within the brain (Steinmetz et al., 2019); in fact, researchers have recorded up to 10,000 neurons in a 0.3 mm^3^ portion of the brain using two-photon imaging (Stringer et al., 2019). (B). Stimulus encoding can occur at the single neuron level, where, for example, it could encode the direction of a stimulus. Stimulus encoding can also occur at the population level, where multiple units together can provide a better representation of the stimulus, including its direction and color. (C) Example of populations of neurons responding to the direction of water flow along the fish’s tail (Vanwalleghem et al., 2020). These neuronal populations show a tonic activity for the duration (10 s) of a water flow stimulus from the tail to the head (green arrow), or from the head to the tail (magenta arrow). The neurons were clustered in three categories: bidirectional response (blue), head to tail (pink), or tail to head (gold). Here we represent their spatial coordinates in the zebrafish brain (left). Telen = telencephalon, OT = optic tectum, TS = torus semicircularis (small dotted lines), ON = octavolateralis nucleus. (D) It is possible to predict neuronal activity from behavior, which in this example is the facial movement of a mouse. (Top) Motion energy was computed from consecutive frames (*t* and *t* + 1). (Middle) The principal components could then be used to predict the neuronal activity of 1,000 neurons. (Bottom) The predicted activity was remarkably similar (about 30%) to the real neuronal activity (sorted with an embedding for visualization). Adapted from Stringer et al. (2019).

How can we make sense of the brain? Researchers can now record the activity of thousands of individual neurons across the whole brain (Figure 1A) thanks to methodological improvements in multielectrode arrays (Branner & Normann, 2000; Paulk et al., 2022; Steinmetz et al., 2019), and calcium indicators combined with fast microscopy (Aimon et al., 2019; T.-W. Chen et al., 2013; Hastings et al., 1969; Kim et al., 2017; Miyawaki et al., 1997; Nakai et al., 2001; Nguyen et al., 2016; Stringer et al., 2019; Tsien et al., 1982; Vanwalleghem et al., 2018). With these new tools in hand, the number of recorded neurons has doubled every seven years since 1960 and, as a consequence, neuroscience is relying increasingly on “big data” approaches (I. H. Stevenson & Kording, 2011). While the methodological advances that allow researchers to fill the space between individual neurons and the whole brain are exhilarating, the mountains of data behind them present new conceptual challenges to understand them fully.

Systems neuroscience is less interested in the activity, either spontaneous or elicited, of each individual neuron, but more in how ensembles, or populations, of neurons form and interact (Figure 1B). Population coding complements the single neuron encoding approach (Barlow, 1972; Theunissen & Miller, 1995), with a population-level encoding of information by ensembles of neurons (Knight, 1972). This population level of encoding can combine multiple features of a stimulus and ensure the robustness of a behavior despite individual neurons’ stochasticity and noise (Quian Quiroga & Panzeri, 2009). Of course, population-level encoding can only be described and analyzed if one can record from multiple neurons simultaneously.

A number of approaches have been developed or adapted for the analysis of population-level data. These approaches include dimensionality reduction (Mu et al., 2019; A. W. Thompson et al., 2016), linear regression (X. Chen et al., 2018; Vladimirov et al., 2018), clustering (Mölter et al., 2018; Vanwalleghem et al., 2020), nonlinear analyses (Ponce-Alvarez et al., 2018), and topological approaches (Gracia-Tabuenca et al., 2020; A. E. Sizemore et al., 2019), to name a few. For many of these methods, the dimensionality and size of population-level datasets can be a limiting factor and require either the use of distributed computing (Freeman et al., 2014), dimensionality reduction (Betzel, 2020), or new mathematical tools (Haesemeyer et al., 2019; Tajima et al., 2015; Triplett et al., 2020; Watanakeesuntorn et al., 2020). Another challenge is the fabled needle in a haystack: identifying a potential sparse population that would drive the animal’s response (Ince et al., 2013; Tajima et al., 2017). Ultimately, all of these tools aim to understand how the brain encodes stimuli and transforms the sensory input into meaningful information and behavior.

Neuronal ensembles encoding sensory stimuli have been observed in zebrafish for all sensory modalities from visual, to auditory and vestibular (Avitan et al., 2020; X. Chen et al., 2018; Favre-Bulle et al., 2020; Favre-Bulle et al., 2018; Friedrich et al., 2004; Migault et al., 2018; Poulsen et al., 2021; A. W. Thompson et al., 2016; Vanwalleghem et al., 2017; Vanwalleghem et al., 2020). For example, the lateral line is a specialized apparatus that senses water flow in fish and amphibians. We showed the existence of neuronal representations of the direction and speed of the water flow (Figure 1C), as well as of the simulated distance traveled by the animal (Vanwalleghem et al., 2020). For visual processing, X. Chen et al. (2018) showed how the transformation of visual stimuli into motor commands was distributed across the brain, before converging onto small clusters of neurons in the hindbrain that output the motor commands. A systems-wide approach was needed to observe the distributed nature of neuronal representations in the above studies and has shown a similar distribution across the brain in mice using Neuropixels probes (Steinmetz et al., 2019) and across the visual cortex using two-photon imaging (Figure 1D; Stringer et al., 2019).

The key limitation of most whole-brain work is its descriptive nature. Studies have linked neuronal responses to stimuli, but few offer testable models or hypotheses on how the sensory processing can drive behavior. Testing the causal link between neuronal responses and behavior is particularly complex when the responses are spatially distributed, as described above. Promising approaches for surmounting this challenge include light-shaping and optogenetics, or ablation, which could allow neuroscientists to interrogate distributed neuronal circuits and identify the necessary parts underlying a specific behavior (Dal Maschio et al., 2017; Dunn et al., 2016; Fernandes et al., 2021; Marshel et al., 2019; Naumann et al., 2016; Vladimirov et al., 2018). For example, Helmbrecht et al. (2018) showed that optogenetic activation of specific regions within the optic tectum could trigger different behavioral programs: an approach program or an escape attempt. The investigators further identified multiple classes of projection neurons from the optic tectum to the hindbrain. These projections could convey the relevant information to the motor centers, including the position of the stimulus. Such a combination of structural and functional data provides a more comprehensive picture of, as well as testable hypotheses regarding, how information flows between brain regions and how that information could be transformed to drive specific behaviors.

Our goal is for this review to be accessible to biologists with little or no mathematical training, since understanding the basis of the most common approaches will empower neuroscientists to choose the most suitable method to answer their specific research question. As most of the methods we discuss involve relations between units, we will begin with approaches for quantifying simple relations between two variables, and from there build up to approaches involving multiple variables such as network science. Finally, we will examine the potential of generative models, and discuss avenues for future research, and how these tools can be used in other contexts. We direct the readers who want to deepen their understanding of a specific topic to these excellent reviews on linear models for encoding (Marinescu et al., 2018; Paninski et al., 2007) and graph theory (Bassett & Sporns, 2017; Sporns, 2018).

## EVALUATING RELATIONS BETWEEN TWO UNITS

**Figure 2.**
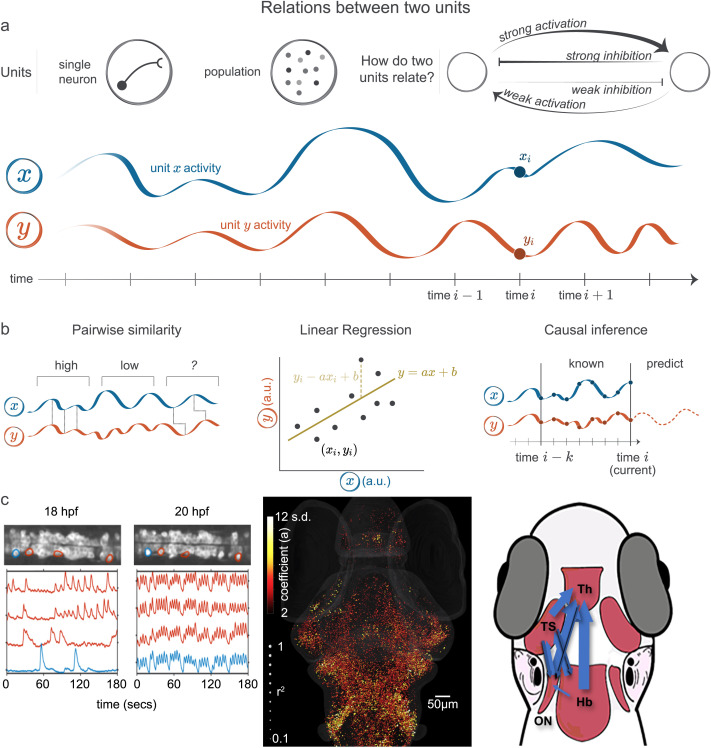
Calculating relationships between two units. (A) In systems neuroscience, a single unit may correspond to a neuron or a population of neurons. Two units may potentially interact in a variety of ways. For example, one can either activate or inhibit the other; the strength of these interactions can also vary, and feedback between units can also exist. As an example, units *x* (blue) and *y* (orange) have activities that change over time. At time *i*, unit *x* has activity *x*_*i*_ and unit *y* has activity *y*_*i*_. (B) Multiple methods exist for quantifying relations between two variables. (Left) Pairwise similarity measures often see the leftmost portion of the activity traces as having high similarity and the middle portion as having low similarity; these measures may vary on their interpretation of the rightmost portion. (Middle) Linear regression calculates a line of best fit (darker gold) given the points (*x*_*i*_, *y*_*i*_) for each time *i*. This line of best fit minimizes the error between the predicted values of *y*_*i*_, using *x*_*i*_, and the true values *y*_*i*_ (lighter gold). (Right) Causal inference methods ask whether information about the activity of unit *x* between time *i* − *k* and the current time *i* improves the prediction accuracy of the future activity of unit *y*, above and beyond the prediction accuracy obtained by using the previous activity of unit *y* alone. (C) Applications of each method to larval zebrafish data. (Left) The pairwise similarity (correlation) between spinal neurons increases dramatically between 18 and 20 hours post fertilization (hpf), as can be seen by the blue and red activity traces moving in synchrony. Adapted from Warp et al. (2012). (Middle) Linear regression was used to quantify the responses to auditory stimuli; the coefficient is mapped to the color of each neuron in the brain, while the coefficient of determination *R*^2^ is mapped to the size of the sphere. Adapted from Poulsen et al. (2021). (Right) The Granger causality approach was used to identify the flow of information between auditory brain regions in Vanwalleghem et al. (2017). Th = thalamus, TS = torus semicircularis, ON = octavolateralis nucleus, Hb = hindbrain.

Systems neuroscience offers the perspective that no one neuron functions alone, visual stimuli influence future motor sequences, a triggered memory may evoke an emotional reaction, and sudden sounds shift attention. All of these examples can be simplified into one unit, for example a neuron or population (see Figure 2A), that influences, connects to, or *relates to* another unit. Determining whether two units are related may seem to be simple at first glance. For example, in which periods of time (Figure 2, x-axis) is the activity time series of one unit tracking the activity of the other unit? Despite the simple question statement, the path to an answer is not immediately obvious, as there are many types of computational methods available from which we must choose. We will highlight a few classical examples, including linear regression and causal inferences, but invite interested readers to peruse Cliff et al. (2022) for a deeper comparison.

### Is the Activity of Two Units Pairwise Similar?

The precise definition of *pairwise similar* has resulted in a multitude of mathematical descriptions that we will not explore here (Bobadilla-Suarez et al., 2020; Miri et al., 2011). At a basic level, the activity of two units being similar might mean that changes in the activity of unit *x* coincide with changes in the activity of unit *y* (Figure 2B). For example, the correlation between spinal neurons drastically increases during the development of larval zebrafish (Figure 2C, left; Warp et al., 2012). The central pattern generator is dependent on this correlated, and contralaterally anticorrelated, activity to generate the rhythmic movements mediating swimming in zebrafish. Importantly there are myriad additional approaches to the problem of evaluating a relationship between two units (Cutts & Eglen, 2014). While no one method is error-free, supporting conclusions with multiple types of pairwise similarity measures from different domains can reduce the effects of bias on the overall outcome.

### How Is the Activity of Two Units Related?

Given the activity of two neurons, we might want to understand *how* they are related. Linear regression can be used to answer this question, by expressing the activity of the second neuron as a linear function of the activity of the first neuron (Figure 2B, middle). When additional variables are present that might affect the results, such as neuron type or location, we can also incorporate these variables into our model to understand or mitigate the influence of such variables on the relationship in question (Smith, 2018). Linear regression is a common but powerful tool that sets the stage for more general approaches such as general linear models and machine learning. In systems neuroscience specifically, these techniques have been used to assess learning (Luchiari & Chacon, 2013; Tompson et al., 2020) and to determine neurons associated with a behavior (Marques et al., 2020; Miri et al., 2011). Linear regression has also been used to assess the response to auditory stimuli in larval zebrafish (Figure 2C, middle). Poulsen et al. (2021) showed that the auditory responses were more widespread than previously observed, but also confirmed previous observations of the primary auditory regions in larval zebrafish (Privat et al., 2019; Vanwalleghem et al., 2017).

### Causal Inference

Knowledge of an association does not necessarily help us with designing intervention strategies, mapping out novel circuits, or determining mechanisms (Nienborg & Cumming, 2010). In order to reach these goals, we need to start with an understanding of causal relationships between neuronal units. For this review, we will say a *cause* of an effect must both precede the effect and provide predictive information about the effect (Cekic et al., 2018; Granger, 1969; Wiener, 1956). This notion of a cause is commonly referred to as Granger causality or G-causality. If unit *x* activity causes a change in unit *y* activity, then given our perspective on causality, we would expect to see that past and present activity of unit *x* can predict the activity of unit *y*, or that unit *x* activity *Granger-causes* unit *y* activity. These ideas support the way many expect the brain to function, and so have been used frequently in neuroscience. For example, G-causality has been used to understand information flow in auditory processing, which confirmed previous results of neuroanatomical studies and aligned with evidence in the mammalian literature (Figure 2C, right; Vanwalleghem et al., 2017) and prey capture (Oldfield et al., 2020) in larval zebrafish. G-causality is one among many causal inference approaches; for example, a method was specifically designed to infer causality using optogenetics by compensating for the inherent confounds (Lepperød et al., 2018). Other methods, such as empirical dynamic modeling, are more appropriate for nonlinear dynamical systems and have been applied to neuronal data (Watanakeesuntorn et al., 2020).

In summary, there are many ways to test for the presence of a relationship between two interacting units, and while the methods we discussed have been useful for systems neuroscience, it is important to choose the approach based on the data, the system, and the specific question at hand (Torres et al., 2020).

## EVALUATING PATTERNS OF MANY INTERACTING UNITS

**Figure 3.**
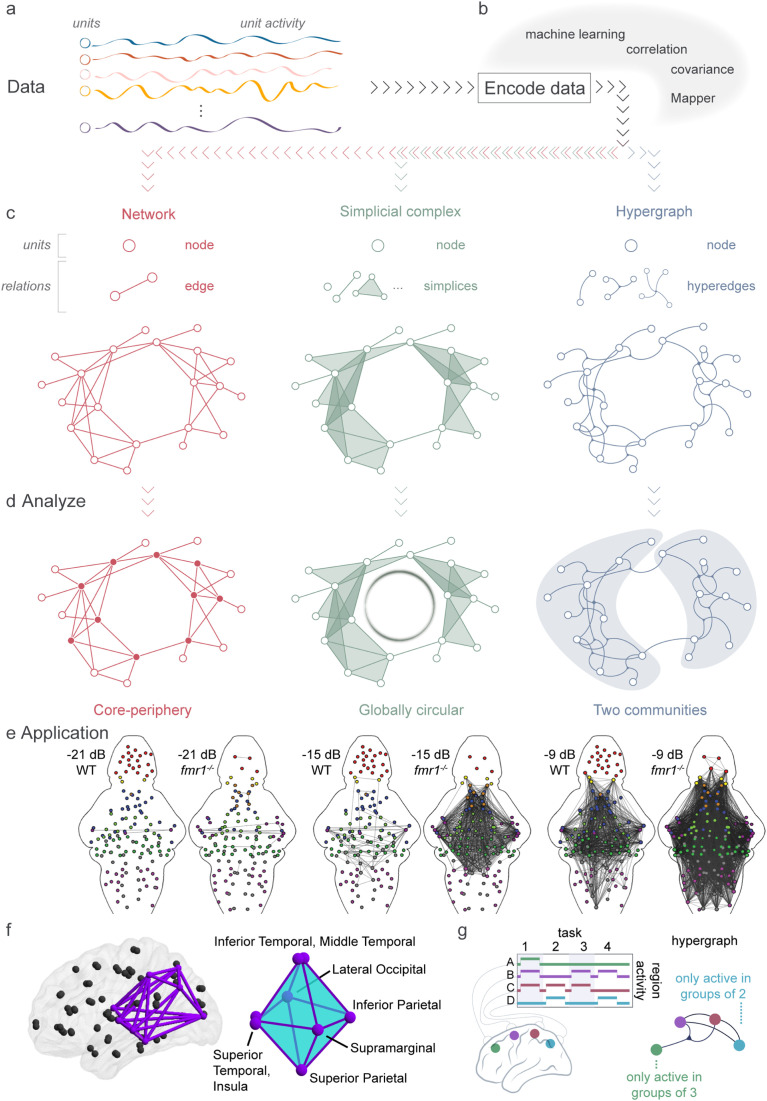
Systems neuroscience uses multiple methods to encode, represent, and analyze data. (A) Data consists of many units with recorded activity over time. (B) There are many options for encoding data into a mathematical representation. (C) Examples of representations. (Left) A network is composed of nodes and edges that connect exactly two nodes. (Middle) A simplicial complex is constructed from nodes and simplices that connect any number of nodes, and any subset of connected nodes. (Right) A hypergraph is built from nodes and hyperedges that connect any number of nodes. (D) Based on the type of representation used (network, simplicial complex, hypergraph), different analysis approaches become available. For example, representing data as a network allows one to detect many types of structure, and more recently very specific patterns such as core-periphery organization. Representing data as a simplicial complex permits the use of homology, which perceives the circular nature of the complex. Finally, a hypergraph representation enables a unique perspective on the community structure of the system. While one could use any of these representations to calculate, for example, community structure, we stress that each representation will provide a unique perspective on community structure, and furthermore that some representations are more amenable to particular analyses, for example, finding loops, than others (Torres et al., 2020). (E) Application of network theory to larval zebrafish data. WT and *fmr1*^-/-^ larval zebrafish were presented with increasing intensity of sound, from −21 dB to −9 dB, and their neuronal activity was recorded. Circle plots of the edges between brain region nodes in WT and *fmr1*^-/-^ zebrafish; an edge was placed if the correlation between their response to auditory stimuli is above 0.85. Node color indicates brain regions, in order: telencephalon, red; torus semicircularis, dark magenta; cerebellum, dark green; thalamus, orange; hindbrain without the Cb and ON, gray; octavolateralis nucleus, magenta; pretectum, light blue; optic tectum, blue; habenulae, yellow; tegmentum, light green. Adapted from Constantin et al. (2020). (F) Application of topological data analysis to the structural connectome. Example conserved cavity connecting visual processing regions shown in the brain (left) and abstracted for ease of interpretation (right). For visual simplicity, the cavity depiction in the brain does not include shading for 2-simplices, only edges. Adapted from A. E. Sizemore et al. (2018). (G) A hypergraph created from recordings of the on/off activity of brain regions during four tasks reveals that the green node *A* is only active in groups of three regions, whereas the blue node *D* is only active in groups of two regions. As such the green, magenta, and red nodes are all linked by a hyperedge. Adapted from Torres et al. (2020).

Instead of focusing on only one pair of variables, we can expand our scope to include many interacting units such as thousands of neurons or hundreds of neuronal populations (Figure 3A). Approaches under the umbrella of complexity, or network, science mathematically aim to encode the data as units and parts (interactions, relations) of a quantitative object, such as a network, then use computational methods to evaluate the representation and make predictions about how that structure influences biological function. Below we draw mainly from the two subfields of network neuroscience and topological data analysis, but we invite the interested reader to peruse Battiston et al. (2020), Spivak (2009), and Torres et al. (2020) for deeper discussions.

### Encoding Data

The first step is to decide what the units, or nodes, will be, either individual neuronal units, or nodes representing multiple neuronal units, such as those that respond to the same stimulus (Figure 3A; Crossley et al., 2013). The choice of encoding method, which takes data and creates from it a quantitative object (Figure 3B), is crucial to the success of the entire analysis. If chosen incorrectly, it can cause unexpected results, bias the computations, and prevent the analysis from answering the initial question (Torres et al., 2020). Finally, we must decide what will constitute a relation between units. As discussed above, one could use *pairwise similarity*, or *causal inference* to identify relations between any pair of nodes (Fallani et al., 2015; Watanakeesuntorn et al., 2020). Using these ideas, one can construct a graphical model in which a relation between two units indicates a pairwise dependence. Such models are common in neuroscience (Belilovsky et al., 2015; Chang et al., 2019). Additionally, techniques from machine learning can provide a whole-brain approach to creating networks (Murugesan et al., 2020). Finally, one can use probabilistic graphical models to represent neuronal ensembles and identify neurons that can activate the whole ensembles (Carrillo-Reid et al., 2021).

Many of the above discussed methods use data only from the two units in question, but when encoding data involving many units, we can use methods that incorporate all of these data. For example, Bayesian networks (Bielza & Larrañaga, 2014; Javidian et al., 2020) provide a representation of the joint probability distributions across all the units within the system, so that a relation indicates dependence of the target on the source. Finally, a method called Mapper creates a representation from high-dimensional data by forming nodes from (overlapping) clusters of data points and relations between nodes with corresponding clusters that share data points (Cámara, 2017; Singh et al., 2007). Mapper’s representation not only offers a new way to visualize high-dimensional data, but additionally creates a computational object (network) upon which one can perform downstream analyses such as assessing community structure of a Mapper representation created from fMRI data (Saggar et al., 2018).

Importantly, the type of object constructed from the data-encoding process can be constrained by the method of encoding. Let us consider three mathematical frameworks with which we could represent our data (Figure 3C). The first is a network, which has units (nodes) and exclusively pairwise relations between nodes. The second and third, simplicial complexes and hypergraphs, respectively, allow for polyadic relations among nodes, meaning that any number of nodes can be related. Taken together, in choosing the encoding strategy it is crucial to consider (a) how to define nodes, (b) what a relation between nodes should capture, and (c) the frameworks that can be created with the approach. It is then important to ensure that all of these fit within the larger research question and biological system.

### Network Science

By representing data as a network composed of nodes interconnected by edges, the investigator can answer new questions outside the reach of analytical techniques that focus on pairwise interactions (Bassett et al., 2018). Specifically, one can ask questions about how the full pattern of relations—the network—might relate to the system’s dynamics, the associated processes of perception, action, and cognition, and the resultant behavior (Fornito et al., 2016). Across many networks, the values of related measures are correlated; notably, however, the converse is not necessarily true (da Fontoura Costa et al., 2007). The values of mathematically unrelated measures can still be correlated across a network ensemble if they are being simultaneously driven by a single mechanism (Onnela et al., 2012; A. Sizemore et al., 2016). Relatedly, some statistical measures might vary across an ensemble in a way that tracks variation in health, severity of disease, type of computation, and accuracy, complexity, or form of behavior.

One dimension along which network measures range is the dimension of scale, from local, as typically thought to be reflected in the clustering coefficient, to global, as typically thought to be reflected in the shortest path length (Bullmore & Sporns, 2009). Networks that have both high clustering (similar to a lattice) and low shortest path length (similar to a random network) are said to have small-world organization (Bassett & Bullmore, 2006, 2017; Muldoon et al., 2016). Evidence suggests that the network of neurons in zebrafish larvae has a small-world organization (Stobb et al., 2012), suggesting its capacity for both local information processing and global information transmission. This organization is also seen in *C. elegans* and other species across diverse evolutionary lineages (Kaiser & Varier, 2011). However, it may be that this small-world organization only holds at certain scales (synapses, circuits, brain areas, etc.), or depends on the empirical approach taken to gather and analyze the data (Hilgetag & Goulas, 2016; Muller et al., 2014). For example, scale-free networks, where the degree distribution follows a power law, have also been observed in the optic tectum of larval zebrafish (Avitan et al., 2017), and biological networks as a whole have been found to exhibit strong to strongest evidence of scale-free structure in 12% of the networks studied (Broido & Clauset, 2019). It will be important for future generative models to incorporate additional factors beyond small-worldness, such as the importance of long-term connections to the functional diversity of distant brain areas, and not only topological considerations (Betzel & Bassett, 2018).

In the middle of the continuum from local to global structure in networks is the mesoscale (Figure 3D); for example, a network is said to have *core-periphery* structure if it contains (a) a core, which is a set of densely interconnected nodes, and (b) a periphery, which is a set of nodes that connect to the core, but do not tend to connect to other nodes in the periphery (Rombach et al., 2014). It is important to note that the local and global measures discussed above are independent of the structure. Notably, mesoscale structure has been shown to vary across cognitive states (Pedersen et al., 2018) and stages of learning (Bassett & Mattar, 2017), and to be altered by genetic risk for psychiatric disease (Dimitriadis et al., 2021). These mesoscale structures have been observed in *Drosophila* (Shih et al., 2015; Shih et al., 2020); functional connectomes display hierarchical modularity that reconfigures appreciably in response to a stimulus in larval zebrafish (Betzel, 2020). Constantin et al. (2020) showed that the zebrafish model of fragile X syndrome had increased sensitivity to sound, especially apparent in the higher connectivity across brain regions at lower sound intensity (Figure 3E, −15 dB). Such an increased connectivity is consistent with one of the theories developed to explain the basis of the sensory phenotype in fragile X syndrome (Testa-Silva et al., 2012). For a deeper dive in the use of networks with calcium imaging data, we invite readers to consider Nelson and Bonner (2021).

### Topological Data Analysis

Methods from topological data analysis (TDA) aim to provide a quantitative and qualitative description of how the pieces of the system combine to form the larger whole (Blevins & Bassett, 2020; Geniesse et al., 2019; Ghrist, 2007; Munch, 2017). This topological perspective deviates from many familiar frameworks, such as principal component analysis, by requiring no a priori assumption about the space of the data (for example, that the data are linear). Similar to network science, TDA can represent systems with a simplicial complex, which contains nodes (units) and simplices (parts or interaction representations, see Figure 3C). While the edge of a graph can connect only two nodes, a simplex can connect any number of nodes. For example, three nodes can be all together connected by a simplex (illustrated as a filled triangle in Figure 3C); specifically, a *k*-simplex connects *k* + 1 nodes in a simplicial complex. In Figure 3C, we see that the simplicial complex has simplices drawn as links (1-simplices), shaded triangles (2-simplices), and shaded tetrahedra (4-simplices), and thus can represent many more types of multi-node interactions than the network. Encoding such polyadic relations between nodes provides TDA with distinct advantages, such as the ability to represent many-unit neuronal interactions and the means to detect system features not perceived in other domains. If we examine three neurons responding to different stimuli, the simplicial complex lets us represent the difference between a system in which every pair of neurons co-fires in response to different stimuli, and a system in which all three neurons fire in response to the same stimulus. Though we here have presented only a brief introduction to these TDA ideas, we direct the interested reader to Ahissar and Kleinfeld (2003), Blevins and Bassett (2020), Lisman and Grace (2005), Ma’ayan et al. (2008), Munch (2017), A. E. Sizemore et al. (2019), and Tallon-Baudry et al. (2001).

Loops within a neuronal system can support memories (Lisman & Grace, 2005; Tallon-Baudry et al., 2001), permit computations (Ahissar & Kleinfeld, 2003), and affect dynamics (Ma’ayan et al., 2008). We can detect such looped motifs by using homology to detect loops that surround topological cavities or voids within the simplicial complex representation of the system (for example, the large looped nature of the simplicial complex in Figure 3D). Specifically, loops that surround a cavity would intuitively allow for two routes of information to travel from one side of the loop to another, or could allow a signal to cyclically continue around the loop and thus repeat periodically. Furthermore, homology, and its weighted counterpart, persistent homology, can detect cavities, and thus loops, of any dimension within the system. For example, Reimann et al. (2017) identify high-dimensional loops within the correlated activity of neurons, and find that in general such topological complexity depends upon neuron morphology. Additionally, A. E. Sizemore et al. (2018) found loops connecting cortical to subcortical regions in the structural connectome to be a consistent feature across healthy adults. Shown in Figure 3F, one of these conserved loops connects higher order visual processing regions and mimics portions of the dorsal and ventral visual streams. The conserved nature of these loops found across individuals, but not in null models, suggests their importance in computation (A. E. Sizemore et al., 2018). Additionally, these homological ideas have improved our understanding of neuronal dynamics (Bardin et al., 2019; Curto, 2016; Curto et al., 2012), stimulus encoding (Basso et al., 2016; Giusti et al., 2015; Rubin et al., 2019), and the time-evolving brain (Yoo et al., 2016).

Although we introduced Mapper as a method for encoding data, it has also been used as a stand-alone tool to understand neural systems as it allows one to represent large datasets in a reduced form. Simply having the ability to visualize high-dimensional data using the Mapper algorithm has led to the discovery of a new breast cancer subgroup (Nicolau et al., 2011) and has been used to predict drug effects in traumatic brain injury (Nielson et al., 2015). Additionally, Saggar et al. (2018) paired Mapper with functional imaging data to investigate features of an individual’s brain state space. Their work led to the creation of DyNeuSR (Geniesse et al., 2019), a Python implementation of Mapper designed specifically for neuroimaging. With these implementations of Mapper, researchers can get a graphical representation of brain states, for example, observe how individuals traverse across the graph (brain states), and from this sequence better understand state transitions between different cognitive tasks (Geniesse et al., 2022).

### Alternative Representations

Not all neural systems are most faithfully represented by networks or simplicial complexes. Many other representations exist, including but not limited to hypergraphs, higher order networks, multilayer networks, and sheaves, which we will all briefly describe below. Similar to simplicial complexes, hypergraphs can encode polyadic relationships among nodes (in Figure 3C, comparing simplices to hyperedges). However, whereas a 2-simplex (a shaded triangle) implies that all pairs of nodes are pairwise related, a hyperedge connecting three nodes does *not* imply that any pair of the three nodes is pairwise related. This feature allows the hypergraph to represent a wide variety of systems, such as functional (Davison et al., 2015; Davison et al., 2016) and structural neural systems. For example, after recording neural responses across different tasks, we could create a hypergraph in which brain regions are connected by a hyperedge if they are coactive in a task (Figure 3G). For example, the three regions *A*, *B*, *C* are all coactive in a task (Figure 3G, task 1), but regions *A* and *B* are never coactive in any other task without *C*. Detecting many large hyperedges (hyperedges that connect many nodes) indicates that often many populations act in unison in the tasks. Furthermore, if we find many large hyperedges but few small hyperedges, then we could conclude that generally these populations function only alongside others, and rarely by themselves or in small groups. Such a phenomenon can be quantified by the hypergraph-specific measurement called the *fill coefficient* (Torres et al., 2020).

Higher order networks (Xu et al., 2016) are designed to handle data from paths, where the activity of a node could depend on multiple previous events, such as from a sequence of visited brain states where the current brain state does not depend on only the one immediately preceding it. For example, in Figure 3G one could imagine a situation in which the response of region *B* to task 4 would depend on the responses of region *D* not only in task 2, but also in task 1. Such a situation would show a second-order dependency of *B* as it depends on the state of *D* two steps back. Multilayer networks represent systems that might (a) have different types of edges such as correlations in different frequency bands (Buldú & Porter, 2018), or different data types entirely; (b) evolve with time (W. H. Thompson et al., 2017); or (c) include multiple subjects (Muldoon & Bassett, 2016). For example, one could imagine a multilayer network where each node is a neuron and the edges in each layer would represent (a) structural connectivity, (b) functional connectivity, or (c) synapse types and numbers. These multilayer networks offer an attractive option to integrate multiple types of data, as reviewed in Boccaletti et al. (2014) and discussed later. Additionally, sheaves are a mathematical framework that allows one to represent not only the connections between nodes (as in the networks above), but also the specific relationship, such as linear maps, between node activities or properties that these connections represent. Thus far sheaves have been used for network coding problems (Ghrist & Hiraoka, 2011), signal processing (Robinson, 2013), and other applications (Phan-Luong, 2008), but they have great potential for use in biological research (Blevins & Bassett, 2020; Hansen & Ghrist, 2020). Though certainly not complete, we offer the above examples to show the seemingly limitless possibilities of mathematical system representations (Battiston et al., 2020; Torres et al., 2020).

## EVALUATING MECHANISMS USING GENERATIVE MODELS

By encoding data as a set of pairwise interactions, a network, or a simplicial complex, investigators can use statistical and computational techniques to describe the patterns they see. However, description does not amount to explanation, and nor does a measurement automatically produce an understanding. To move beyond description and measurement, and toward explanation and understanding, it can often be useful to define generative models (Betzel & Bassett, 2017a). With what ingredients and assumptions could one generate the observed pairwise interactions? Or the network? Or the simplicial complex? If one were able to devise a set of rules that produced data similar to those observed, then one would have identified a candidate mechanism for the observations (Bertolero & Bassett, 2020). Generative models do just that. A generative model is a set of interaction rules that produces an outcome; and hence, generative modeling as an approach can be used to determine what candidate mechanisms may have produced the observed data. Although generative models exist at all scales of analysis, here we will focus on generative models for networks. For those readers interested in other scales, see Kanashiro et al. (2017) for a cellular-scale generative model of interactions and Sizemore Blevins and Bassett (2020) for generative models of simplicial complexes.

Generative network models are defined by the specification of a wiring rule. Typically, wiring rules are defined probabilistically. For example, a node has a probability of connecting to other nodes with certain features. In brain networks, two features are commonly evaluated: (a) a geometric feature such as the physical distance between two nodes, and (b) a topological feature such as the degree or clustering coefficient of a node. For example, one might generate a network in which the probability of connecting node *i* to node *j* is negatively associated with the distance between them and positively associated with the product of the degree of node *i* and the degree of node *j*. The geometric and topological factors formally encode the two competing drivers of connectivity: wiring minimization and topological efficiency (Bullmore & Sporns, 2012). A minimally wired network, in which only the shortest possible links are present, would contain only edges between nearby nodes, requiring inefficient long relays to get information from one part of the network to another. Conversely, a maximally efficient topology, one in which topological path lengths are short, would contain edges between randomly chosen pairs of nodes, requiring costly long-distance tracts. By balancing these two competing pressures, the two-feature generative models are able to produce networks with topologies that are notably similar to those observed in real neural systems.

Generative network models are fairly young, and therefore a relatively small literature exists applying them to open questions in systems neuroscience. Yet despite their youth, generative network models have already been considered across species from *C. elegans* (Nicosia et al., 2013) to humans (Betzel et al., 2016), from cellular connectomes (Vértes et al., 2014) to macroscale connectomes (Betzel et al., 2016), and from healthy states to diseases states (Vértes et al., 2012; X. Zhang et al., 2021). Efforts have begun extending generative network models from the probabilistic so-called single-shot models that we have just described to growth models; these extensions pose the question of what growth rule (rather than what static wiring rule) might explain the observed trajectories of development, aging, or disease progression. A particularly exciting endeavor along these lines is the examination of a network morphospace constructed by the differential or combined expression of *N* network features (Avena-Koenigsberger et al., 2014; Avena-Koenigsberger et al., 2015). The approach allows the investigator to define a given network starting point (e.g., an infant connectome, or a healthy connectome) and rewire it to maximize or minimize a predefined set of network features, using a multi-objective evolutionary algorithm. The goal is to ask whether that rewiring rule can take the network to a final target point (e.g., a young adult connectome, or a connectome characteristic of a decade or more of neurodegeneration), precisely along the trajectory shown by development or by disease progression (Tang et al., 2017). Such efforts offer the exciting potential to test network hypotheses in a much more formal way than was hitherto possible, and to either validate or disprove posited mechanisms.

## DISCUSSION

The methods described above focus on the functional (in the sense of the inferred function of the neurons) and temporal data from calcium imaging, but an additional challenge will be to integrate those analyses with the multimodal information that can be collected in parallel with calcium imaging. The additional modalities can be the physical position of the neurons (Betzel, 2020; X. Chen et al., 2018; Chiang et al., 2011), their ultrastructure (Hildebrand et al., 2017; Vishwanathan et al., 2022; Zheng et al., 2018), molecular subtype (Lovett-Barron et al., 2017; Shainer et al., 2022), morphology (Kunst et al., 2019), or even other cell-signaling reporters (Linghu et al., 2020). The data from these various modalities can be categorical (subtypes of neurons, morphology), time series (other cell signaling reporters), or numerical values (position and projection trajectory), each potentially coming with their own metrics and specific questions. The Euclidean distance between neurons’ spatial locations is easy to quantify, for example, but it is less easy to know how to integrate these types of data, alongside functional data, in the networks, or hypergraphs, and how to weight each modality to construct the model.

Despite the difficulty of the question of how to integrate multimodal datasets, researchers from many disciplines have pushed forward in this direction. First, multilayer frameworks offer one solution to incorporating multimodal data into one quantitative object. For example, multilayer networks can combine multiple types of neuron connectivity (Schroeder et al., 2022), activity from multiple frequency bands (Yu et al., 2017), or even data across individuals (Vaiana & Muldoon, 2020) within one object. Notably, the multilayer approach is not restricted to networks. Indeed, one could encode data as a multilayer simplicial complex or hypergraph following a similar procedure. Alternatively, we can incorporate data about each node using a node-annotated version of the above frameworks. In an annotated or attributed network, each node is also associated with a category (Bothorel et al., 2015; Yang et al., 2013) or a continuous value. These extra data are then incorporated into analytical methods, for example in performing community detection using task-based fMRI data (Murphy et al., 2016). Extending these ideas naturally leads one to matrix-valued graphs (Trinh et al., 2018) and sheaves (Robinson, 2017), which incorporate additional quantitative information on the relations between nodes and node activity. Still, as we previously discussed, no one framework fits every biological system and experiment, so we leave the finding of the perfect framework as an open question, as there is still incredible space for creative solutions.

Though much of this work has focused on systems composed of individual neurons, an additional future direction might consider how complex systems of neurons connect to that of the entire brain (Betzel & Bassett, 2017b). For example, networks could be created from data collected at the neuron level, population level, and the whole-brain level. In order to handle these multiple scales, one approach would create one network including all data, and use hierarchical methods or community detection to find subnetwork structures and determine how they fit together (Fortunato, 2010; Meunier et al., 2009). Alternatively, one could create a multilayer network with layers corresponding to the different neural scales (Betzel & Bassett, 2017b). Such a multilayer approach could even incorporate multiscale temporal data as well (Betzel & Bassett, 2017b). Networks of networks are even possible and often studied under the name of interdependent networks using a multilayer network framework (Gao et al., 2011). In TDA, the mathematics behind the methods allow for simple deconstruction of topological motifs of small complexes that are sub-complexes of a larger simplicial complex, thus allowing these tools to naturally incorporate multiscale data (Yoon & Ghrist, 2020). Finally, at a more fundamental level one can control the resolution using the definition of nodes. For example, one might define a node based on one neuron, a group of neurons, or even similarly clustered data (Betzel, 2020; van Veen et al., 2019).

As described above, graphs and network science may provide a common framework in which to integrate multiple information modalities (function, position, and neuronal identity, among others), and multiple scales of information. Notably, graphs can incorporate changes across time and represent the changing dynamics of the brain in different circumstances. The visualization and analytical tools offered by network science can look beyond pairwise interactions and reveal larger patterns of interactions or dynamics. As such, graphs provide a powerful basis on which to generate models of information processing and flow. Based on the observed network statistics, one can test a set of wiring rules and evaluate whether it generates a model similar to the observed network. Growth rules can be added to modify the wiring of the model across various trajectories such as development or disease. These rules can provide testable hypotheses on the mechanism underlying the observed dynamics of the network. Overall, the best test of any model is in its prediction accuracy, combined with advances in causal inferences, and circuit disruption technologies. One can imagine a scenario where you can predict how your model would react to a given perturbation of its wiring, and test it in vivo.

Finally, while this review focuses on systems neuroscience, these methods can be used in any quantitative biological context. In ecology, one can use causal inference to identify how species affect each other (Sugihara et al., 2012; D. D. Zhang et al., 2011) or network theory approaches to model food webs or spatial interactions (Casselberry et al., 2020; Krause et al., 2003). The same applies to cell biology, which benefited immensely from network approaches for characterizing regulatory gene networks and protein-protein interactions, among other interactions (Aittokallio & Schwikowski, 2006; Horvath & Dong, 2008; Martin et al., 2010). With the expansion of calcium imaging into nonneural tissues, the tools discussed here will also provide additional opportunities to better quantify, model, and ultimately understand the role calcium and intercell communication may play in diverse developmental and physiological processes (Balaji et al., 2017; A. J. Stevenson et al., 2020).

The availability of these types of data and their compatibility and searchability, as well as innovative visualization techniques, will all influence how easily the integration can occur (Akil et al., 2011). Efforts such as those led by the International Neuroinformatics Coordinating Facility (BRAINS Expert Working Group et al., 2017; Eglen et al., 2017) exist to standardize and unify neuroscience datasets, but they have not yet been embraced by the community at large, and we still need to ensure the code can be reusable (Riquelme & Gjorgjieva, 2021). Such commitments and infrastructural resources could serve to increase the power of the analysis of such data, and help to ensure the reproducibility of the research.

## CONCLUSION

We have compiled and explained several of the most common data analysis approaches available to systems neuroscientists. As we show, no single method is superior to all the others and several are complimentary; researchers need to understand each method’s limitations to choose the most appropriate one for their specific question. Knowing which analysis will be used, and its limitations, can also guide experimental design to ensure that the chosen method can be used to its full potential on the dataset. For example, tools that can uncover high-dimensional links, such as hypergraphs, would allow for more complex stimuli or behaviors to be tested. On the contrary, they may require more parameter tuning, or have more built-in assumptions, which may generate spurious results with low-dimensional experiments. We also suggest that the complex systems field may provide robust frameworks with which to integrate multiple streams of information. For example, multilayer networks, or networks of networks, could represent different information modalities (functional imaging, molecular identity, physical location, or others) or different scales (subcellular, single cell, populations, or whole brain). These frameworks also benefit from the numerous tools that the wider mathematics community has developed. In conclusion, we propose that multilayer networks are our best tool to integrate multimodal information into a single framework.

## CITATION DIVERSITY STATEMENT

Recent work in several fields of science has identified a bias in citation practices such that papers from women and other minority scholars are under-cited relative to the number of such papers in the field (Caplar et al., 2017; Dion et al., 2018; Dworkin et al., 2020; Maliniak et al., 2013; Mitchell et al., 2013). Here we sought to proactively consider choosing references that reflect the diversity of the field in thought, form of contribution, gender, race, ethnicity, and other factors. First, we obtained the predicted gender of the first and last author of each reference by using databases that store the probability of a first name being carried by a woman (Dworkin et al., 2020; Zhou et al., 2020). By this measure (and excluding self-citations to the first and last authors of our current paper), our references contain 7.05% woman (first) / woman (last), 7.68% man/woman, 15.18% woman/man, and 70.1% man/man. This method is limited in that (a) names, pronouns, and social media profiles used to construct the databases may not, in every case, be indicative of gender identity and (b) it cannot account for intersex, nonbinary, or transgender people. Second, we obtained the predicted racial/ethnic category of the first and last author of each reference by databases that store the probability of a first and last name being carried by an author of color (Ambekar et al., 2009; Sood & Laohaprapanon, 2018). By this measure (and excluding self-citations), our references contain 12.61% author of color (first) / author of color (last), 16.13% white author / author of color, 22.15% author of color / white author, and 49.11% white author / white author. This method is limited in that (a) names and Florida Voter Data to make the predictions may not be indicative of racial/ethnic identity, and (b) it cannot account for Indigenous and mixed-race authors, or those who may face differential biases due to the ambiguous racialization or ethnicization of their names. We look forward to future work that could help us to better understand how to support equitable practices in science.

## AUTHOR CONTRIBUTIONS

Ann S. Blevins: Conceptualization; Visualization; Writing – original draft; Writing – review & editing. Dani S. Bassett: Conceptualization; Supervision; Writing – review & editing. Ethan K. Scott: Supervision; Writing – review & editing. Gilles Claude Vanwalleghem: Conceptualization; Visualization; Writing – original draft; Writing – review & editing.

## FUNDING INFORMATION

DANDRITE, Lundbeckfonden (https://dx.doi.org/10.13039/501100003554), Award ID: DANDRITE-R248-2016-2518. Gilles Claude Vanwalleghem, Novo Nordisk Fonden (https://dx.doi.org/10.13039/501100009708). Gilles Claude Vanwalleghem, Aarhus Universitets Forskningsfond (https://dx.doi.org/10.13039/501100002739). Ethan K. Scott, National Health and Medical Research Council (https://dx.doi.org/10.13039/501100000925), Award ID: APP1066887. Ethan K. Scott, Australian Research Council (https://dx.doi.org/10.13039/501100000923), Award ID: DP140102036 - DP110103612. Ethan K. Scott, Australian Research Council (https://dx.doi.org/10.13039/501100000923), Award ID: FT110100887. Dani S. Bassett, Army Research Office (https://dx.doi.org/10.13039/100000183), Award ID: Bassett-W911NF-14-1-0679 and Grafton-W911NF-16-1-0474.

## TECHNICAL TERMS

Calcium indicators: Fluorescent proteins or dyes whose fluorescence changes with the calcium concentration, typically used as a proxy for neuronal activity.
Lateral line: Sensory organ found in fish and amphibians; it is composed of neuromasts, specialized bundles of hair cells that can detect waterflow.
Optogenetics: Genetic tools that allow the manipulation of cells by light, typically to activate or silence neurons.
Optic tectum: Midbrain region that serves as the main visual processing center in several vertebrates, homologue to the superior colliculus of mammals.
Edge: Connection between two nodes; it indicates they are related, and it can be weighted (measuring the strength of that relation) or unweighted (no information on the strength). It can also have direction or not, depending on the relation metric used.
Degree: Number of connections (edges) from a node to other nodes (and itself).
Fragile X syndrome: Genetic condition caused by the silencing of the *FMR1* gene on the X chromosome (hence the name). The syndrome is linked to intellectual disability in humans.
Traumatic brain injury: Damage to the brain from physical trauma, typically from a blow or impact to the head.
Multilayer networks: These networks allow the interactions of nodes between different layers, each representing different node types, potentially representing different data types.
